# Facing HCV as a Major Public Healthcare Threat in Italy: Epidemiology and Micro-Elimination Pathways among Underserved Populations

**DOI:** 10.3390/healthcare11142109

**Published:** 2023-07-24

**Authors:** Vito Fiore, Valentina Manca, Agnese Colpani, Andrea De Vito, Ivana Maida, Giordano Madeddu, Sergio Babudieri

**Affiliations:** Unit of Infectious and Tropical Diseases, Department of Medicine, Surgery and Pharmacy, University of Sassari, 07100 Sassari, Italy; valentimanca@gmail.com (V.M.); colpaniagnese@gmail.com (A.C.); andreadevitoaho@gmail.com (A.D.V.); imaida@uniss.it (I.M.); giordano@uniss.it (G.M.); babuder@uniss.it (S.B.)

**Keywords:** HCV, underserved populations, incarcerated people, psychiatric disorders, asylum seekers, migrants, refugees, undocumented migrants

## Abstract

Underserved populations have a wide heterogeneity on healthcare provision and use. They also represent the key populations according to WHO 2030 goals for HCV micro-elimination. Our review evaluated the available literature on HCV diagnosis, staging, and treatment among underserved populations, such as incarcerated people, patients with psychiatric disorders, and migrants. A narrative review of literature was performed using key electronic databases (Scopus, Pubmed—MEDLINE) and search engines (Google Scholar). Peer-reviewed publications, grey literature on HCV, and recent models proposed for micro-elimination in underserved populations were included. An insight into the COVID-19 pandemic and its influence on HCV micro-elimination pathways will be also provided. Regarding prison settings, a progressive reduction in HCV epidemiology among incarcerated people in the last years was found (one-third of the level it had been before). People suffering from psychiatric disorders have a high anti-HCV prevalence, but there is a lack of data on active infections. A bidirectional relationship between HCV and psychiatric disorders was found. Migrants showed a very inconsistent assessment of HCV. Furthermore, available studies recorded data from populations with high heterogeneity of anti-HCV prevalence, Therefore, the reported results need caution in their evaluation.

## 1. Introduction

Viral hepatitis C is an infection caused by the hepatitis C virus (HCV). Nowadays, the major way transmission is syringe exchange among people who inject drugs (PWIDs). Rarely, sexual intercourses may be involved [1].

Among infected people, ~50% will develop a chronic hepatitis C (CHC) [2,3,4], with possible severe complications, such as liver cirrhosis and hepatocellular carcinoma (HCC). The recent availability of direct-acting antivirals (DAAs), with short treatments’ schedules and low rates of adverse events (AEs), drastically changed HCV epidemiology and CHC progression to liver cirrhosis and HCC [5]. Moreover, the future patients will be naïve (without previous exposure to older treatments) and will rarely develop cirrhosis, according to the most recent available literature [6].

In Italy, official data from the Italian Ministry of Health reported an anti-HCV prevalence up to 8% [7].

Underserved populations (e.g., incarcerated people, patients suffering from psychiatric disorders, and migrants) represent the most important challenge in HCV diagnosis and treatment [8]. In fact, they have a wide heterogeneity on healthcare provision and use [9]. Furthermore, higher support would be useful to achieve an optimal HCV management in these settings. In fact, a multidisciplinary approach, including and nursing clinical assessment and monitoring, virology, drug and alcohol addiction services, and psychiatric and social support, as well as cultural mediators, is needed [8].

The Italian Ministry of Health funded the introduction of free HCV screening among underserved populations (e.g., PWIDs, incarcerated people) to reach HCV elimination in difficult settings. Tailored interventions are needed to reach these populations’ subgroups [10].

Considering the recent available literature in the field, maintaining the current levels of diagnosis and treatment, ~60% of high-income countries will be late (~20 years) to eliminate HCV as a public health threat, when aiming to meet the WHO’s 2030 targets [11]. As a consequence, creating new, tailored micro-elimination (rapidly tackling HCV in defined subpopulation) pathways for underserved populations is actually crucial.

Our review aims to evaluate the available literature on HCV diagnosis, staging, and treatment among underserved populations. An insight into the COVID-19 pandemic and its influence on HCV micro-elimination pathways will be also provided.

## 2. Materials and Methods

A narrative review was performed, conducting a comprehensive literature search, through key electronic databases (Scopus, Pubmed—MEDLINE) and search engines (Google Scholar), of peer-reviewed publications, grey literature on HCV, and models proposed for HCV micro-elimination among underserved populations, such as incarcerated people, patients suffering of psychiatric disorders, and migrants. The search strategy included the following terms: prison settings, psychiatric disorders, people suffering of psychiatric disorders, migrants, migrant facilities, HCV, and Italy. The literature search had no restriction in timeline or language. Articles were evaluated by title, abstract, and full text. Conferences’ abstracts were also included. After that, duplication between conference abstracts and journal articles was checked. In case of duplication, only the full text was chosen.

## 3. HCV in Prison Settings

### 3.1. Epidemiology

HCV epidemiology has changed incredibly during the last five years, with an anti-HCV prevalence reduction from >30% to <15% in Italian penitentiary institutes, and active infections (HCV-RNA positive) falling from ~90% to ~60%.

A cross-sectional study conducted by Babudieri et al. in 2005 and including 973 incarcerated people, HCV seroprevalence was up to 38% [12]. Four years later, Voller et al. performed a similar survey involving 15,751 individuals. In that case, an HCV seroprevalence of 9% was reported [13]. Sagnelli et al. tested 2241 incarcerated people in 2012, in a program of active case finding. An HCV seroprevalence of 22.8% was found [14].

Data on HCV seroprevalence drastically changed when coming to the DAAs era.

In 2016, Foschi et al. carried out a study among 468 incarcerated people, showing an HCV seroprevalence of 9.8% [15]. In the same year, Babudieri reported data on 5353 incarcerated people, showing an anti-HCV prevalence in 11.3% of included patients [16].

Masarone et al. reported data on 670 incarcerated people. Data were stackable on the previous reported studies, with an anti-HCV positivity ~14% [17].

In 2021, Fiore et al. carried out a multicenter study on HCV in Italian prison settings. In this case, anti-HCV prevalence was ~10%, and active infection in ~41% of cases [18]. These data confirm that the extensive use of direct acting antivirals (DAAs) and fast-track diagnostic approaches have a high impact on HCV control. Furthermore, this type of intervention allows the concomitant diagnosis of other blood borne viruses (BBV). Studies reporting HCV epidemiology and/or treatment in prison settings have been summarized in Table 1.

### 3.2. Clinical Features and Outcomes

The first study focused on liver disease among HCV positive incarcerated people was conducted by Babudieri in 2016 [16]. In that case, the assessment of DAAs eligibility showed how only the 11% of people had a fibrosis, and ~56% had a low fibrosis, according to the FIB-4 score. The most recent literature showed concordance with these data: incarcerated people with CHC are young (~40 years old) and with low liver fibrosis in the majority of cases (up to 96%) [16,18,21]. As a consequence, this is clearly a population which would benefit from strategies with quick testing and staging (with scores and not fibroscan^®^), followed by treatments with short schedules [22].

The literature on HCV treatment in prison settings clearly highlighted a drastic change in terms of outcomes from interferon (IFN) to DAAs era. In 2013, Brandolini et al. reported a cascade of care among incarcerated people receiving interferon. As result, very low treatment rates, and even less SVR rates, were reported [19].

When coming to the DAAs era, the positive treatment outcomes progressively increased in the last years. In a multicenter study from 2018, 142 patients were treated with DAAs, with sustained virological response (SVR) rates of 90.8% [20].

In 2020, an Italian matched cohort showed how SVR rates were not different between incarcerated and non-incarcerated patients, when using DAAs [5]. Moreover, a recent multicenter study on HCV epidemiology and DAAs cascade of care in an Italian prison setting reported even higher SVR rates (~98%) [18]. In this case, the peer-to-peer education, followed by fast diagnostic approach, and the recent wide use of DAAs, drastically changed the HCV epidemiology. This is probably concordant with the concept of ‘treatment as prevention’ used for HAART in HIV.

Actually, a few data are available on HCV among incarcerated women. Studies on viral hepatitis showed a very low rate in women (~2%) [18]. As per official data from the Ministry of Health, women represent <5% of incarcerated population [23]. The only available national study focused on incarcerated women was conducted recently. The Society of Penitentiary Healthcare conducted a survey on 156 women living in prison. As a result, HCV seroprevalence was 20.5% with a 75.5% burden of active infections [24]. Substantially, a very high HCV prevalence was found, demonstrating how the female incarcerated population need more tailored intervention on HCV screening and treatment. However, these data should be confirmed from further studies with larger sample size.

## 4. People Suffering from Psychiatric Disorders

### 4.1. Epidemiology

A few Italian studies have been conducted on the psychiatric population in recent years. Furthermore, the EASL pointed out how the psychiatric population would particularly benefit from a streamlined care pathway. In 2006 Raja et al., published a study including 2396 patients admitted in an Italian urban psychiatric unit. Of them, 42 (2.8%) had anti-HCV positivity [25]. Regarding the socio-demographics influence on HCV prevalence, this was higher among patients with fewer years of education, lower social class, lower last year best Global Assessment of Functioning score, more hostile or violent behavior in hospital, history of previous suicide attempt, and substance-abuse.

A similar study was carried out by De Gennaro et al. In this case, data on people living with HIV (PLWH) with HCV infection and psychiatric disorders were analyzed. The study aim was to assess the treatment efficacy and tolerability in relation to the psychiatric pathology [26]. Among extrahepatic manifestations, neuropsychiatric disorders are frequent among PLWH and HCV infection, with a significant influence on clinical presentation, healthcare use, and outcomes. Psychiatric symptoms such as depression, fatigue, weakness, and anxiety were reported with high frequency in PLWH and HCV infection, causing interference with patient’s ability to perform daily activities, and impairment of quality of life. As result, it can be easy to speculate how there is a bidirectional relationship between psychiatric disorders and HCV, as well as the reverse, which is also the case with PLWH. Recently, Fiore et al. reported data on HCV cascade of care among patients with psychiatric disorders [27]. In this case, 586 people were screened for HCV with quick tests. An anti-HCV prevalence of 39.4% was found in this population, with high rates of active infections (~95%) [27].

Studies reporting HCV epidemiology among people suffering from psychiatric disorders have been summarized in Table 2.

### 4.2. Clinical Features and Outcomes

In 2019, Boglione et al., conducted a survey on 136 patients to analyze the efficacy and safety of DAAs among people affected by psychiatric disorders. In this cohort, patients were affected by schizophrenia (12.5%), bipolarity (16.9%), anxiety disorder (27.2%), behavioral disturbance (15.4%), psychosis (9.6%), and major depression (18.4%). Patients were prescribed by different psychoactive drugs: benzodiazepines (n = 29; 21.3%), antidepressants (n = 24; 17.6%), neuroleptics (n = 29; 21.3%), mood stabilizers (n = 19; 14%), and a combination of drugs (n = 17; 12.5%). SVR12 was observed in 128 patients (94.1%), while 3 (2.2%) were lost in follow-up. No AEs or significant drug-related side effects were reported [29].

The available literature clearly showed how HCV is highly related to psychiatric disorders. This affects the choice of antivirals in relation to the drugs taken for psychiatric pathology.

In the study by Fiore et al., the most common genotypes and clinical characteristics were analyzed; the most common genotype was 3a (98; 44.5%), and patients mostly had a low fibrosis, according with FIB-4 value (142; 64.5%). Of them, one (0.45%) categorically refused the treatment, and one (0.45%) had liver cirrhosis and advanced hepatocellular carcinoma [27]. Overall, 218 patients underwent eligibility for DAAs. The most prescribed treatment was glecaprevir/pibrentasvir (GLE/PIB (172; 78.2%)). The others practiced sofosbuvir/velpatasvir (SOF/VEL). All patients reached the end of treatment. One (0.45%) was lost to follow up, and all the others reached the SVR12.

In the previously discussed study by De Gennaro et al., patients with a documented psychiatric comorbidity were defined as subjects who had received a previous diagnosis by a psychiatric specialist and for whom a psychiatric drug had been initiated. For purposes of analysis, patients were divided based on their psychiatric comorbidity into two groups: subjects experienced with anxiolytic and/or antidepressant (group A) and subjects on treatment with antipsychotic (group B), according to diagnostic criteria of DSM-V [26].

Nineteen patients were included (A:9; B:10). Of the patients, 78.9% were male, mean age was 51 ± 5.9 years, and 31.6% had liver cirrhosis. Four patients (21%) required a change of psychiatric therapy before DAAs initiation. Overall, SVR-24 was achieved in 89.5% of subjects in Intention-To-Treat analysis. Lower SVR-24 rates were observed in those changing psychiatric drugs vs. others (*p* = 0.035). No differences were observed according to antiretroviral treatment change before anti-HCV treatment. At least one mild-to-moderate AEs occurred in four patients (21%). Three severe AEs occurred, leading to two DAA discontinuations [26].

Treatment failure due to the presence of resistance-associated substitutions was observed in three patients. All of them had cirrhosis; two, with genotype 3, were treated with Sofosbuvir (SOF) + Daclatasvir (DAC), and one with genotype 1a was treated with SOF + Simeprevir. Only three subjects did not complete the treatment: two patients stopped the therapy autonomously, and one subject moved to another country for work problems [26].

All HIV/HCV-coinfected patients had an undetectable HIV-RNA at the time of DAAs initiation and seven patients (36.8% of the study population) presented a CDC stage C.

Failure to a previous anti-HCV treatment was reported in four patients (21%), all patients in treatment with anxiolytics and antidepressant (44.4% vs. 0%, *p* = 0.032).

All patients had compensated liver cirrhosis The most frequently prescribed DAA regimen was sofosbuvir + velpatasvir (52.5% overall), especially in subjects in treatment with antipsychotics (80% vs. 33%, *p* = 0.023). In three patients, ribavirin was also added [26].

Dell’ Osso et al. explored instead the worsening or the onset of other psychiatric disorders in patients who are taking eradication therapy with IFN [28].

Overall, 49 patients were included in the study. Of them, 29 (59.2%) were male. The most frequent genotype was genotype 1 (65%) [28]. As result, it was highlighted how for patients treated with IFN who just experienced subthreshold manic-hypomanic symptoms in their lifetime were more likely to develop depression, even without a history of psychiatric disorders [28].

This still confirms how DAAs, which have poor AEs and interactions, represent a breakthrough for HCV treatment in this specific subpopulation.

Data from 2006 published by Raja et al. on urban psychiatric population showed how the 85% HCV included patients had chronic liver-related symptoms, and the 20% still had liver cirrhosis [25]. Moreover, people with severe mental illness had higher risk of HCV infection for a variety of reasons, including elevated rates of injection drug use, multiple, high-risk sexual partners, infrequent use of condoms, a tendency to trade sex for material gain, and engagement in sexual activity while using psychoactive substances [25]. Substantially, this highly underlines the dichotomous relationship between psychiatric disorders and HCV infection [25,27,30,31].

## 5. HCV among Migrants

### 5.1. Migrant Definition and Health Access

Migrant is a widely used term which usually includes asylum seekers, refugees, and economic/climatic migrants. As consequence, it is a term referred to migratory flows to Italy in general. Despite different legal statuses and nationalities, these people typically suffer similar barriers to healthcare provision and use. Italian guidelines recommend offering HCV screening to all migrants from endemic countries (prevalence ≥ 3%) or with risk factors upon arrival [32]. Positive screening results should be confirmed and framed with further analysis, including HCV-RNA and liver enzymes. The patient should be then referred to a hepatologist and evaluated for treatment. Considering that HCV is a chronic disease requiring regular and life-long follow-up visits, the challenge in a highly mobile population is not only the diagnosis, but most important treatment and retention in care.

### 5.2. Epidemiology

Several studies have been conducted in Italy to assess the burden of HCV on the migrant population in the last decade. Included populations are heterogeneous (undocumented migrants, refugees, and asylum seekers), mostly males are enrolled, and follow-up is often unavailable. A recently published prospective study by Colucci et al. reported data from 2019 to 2020 involving 362 migrants (71% male), with high rates of screening acceptance (80%). Six (1.7%) participants tested positive for HCV antibodies and had been in Italy for more than 6 months [33]. The intervention included informational brochures translated into native languages. A similar study was carried out by Prestileo et al. between 2015 and 2017 in Sicily. Undocumented migrants were involved in a screening program, including structured information collection, cultural mediation, and BBV screening. Prestileo recorded a 95.9% acceptance, involving 2751 (72% men). Among them, 24 (0.9%) were found positive for HCV screening, and 18 of them had detectable HCV-RNA. In 2012–2013, Coppola et al. conducted a prospective study including counselling, screening, and a questionnaire regarding risk factors in Naples [34]. They reached a 95.2% acceptance, with 996 people involved (72.3% male). A 4.5% of positive HCV screening was registered among undocumented migrants, while 2.7% among asylum seekers; a total prevalence of 4% was recorded [35]. Several other retrospective studies report a prevalence of HCV between 0.85% and 6.2%. The lowest prevalence reported refers to a group of 118 asylum seekers screened in Verona in 2014–2015 [36], while the higher prevalence was recorded in a multicenter study conducted in 2010–2013 in 46 Italian centers, including 3421 migrants, with a significative presence of East-European migrants among positive (48%, N = 94) [35]. Other studies suggest that a higher prevalence is found among not-recently-arrived migrants. Two studies conducted among African asylum seekers report similar prevalence (Fiore et al., 2019, 1.1% and Donisi et al., 2015, 1.9%) [37,38]. At the same time, two studies addressing undocumented migrants report a higher prevalence, ranging from 2.2% to 3.3% [39,40].

Several studies focusing on specific subpopulations of migrants in Italy were found. In 2000–2015 Prestileo et al. conducted a prospective study in Palermo, addressing incarcerated migrants (Africans and Eastern Europeans) to guarantee equal health access during their stay in prison and after. One-hundred-thirty-three participants were involved. Of them, only one was female and seventy (52.6%) were PWID. Fifty-four tested positive, twenty-five underwent the full diagnostic process, and twenty received a full therapy course, with only one loss to follow-up [41].

Few data regarding migrant women are available. Laganà et al. collected data from migrant women (Africans, Asians, Eastern Europeans) admitted to their service between 2003 and 2013 [42]. All pregnant women were offered a BBV and syphilis screening, for a total of 320 women screened with a prevalence of 0.9% of anti-HCV. Unfortunately, the legal status of the subjects was not reported [42]. However, data from the Italian PITER cohort (Italian platform for the study of viral hepatitis therapy) report a higher number of positive females among migrants when compared to natives [43]. Thus, the burden of HCV on female migrants deserves further studies and should better define epidemiology according to Nationality.

Regarding unaccompanied minors, a study was conducted in Rome between 2013 and 2019. Eight-hundred-and-seventy-nine children from Africa, Asia, and Eastern Europe (98% male) were included [44]. Marrone et al. reported 836 tests, of which 9 were positive (1.1%) and 6 showed detectable HCV-RNA (0.72%) [44]. This may seem a low prevalence, but it is surprisingly high considering that HCV is a chronic infection usually affecting young adults and PWID. No data regarding risk factors were reported; however, vertical transmission should be considered in children coming from endemic countries with low treatment rates.

Other interesting data come from literature regarding migrant sex workers. There are a few studies on sex workers in Italy. We found four Italian studies focusing on sex workers; in 2017, Lapadula et al. conducted a prospective intervention in Milan and Monza, reaching 130 sex workers, with 66 accepting the screening and no positive results [45]. Older studies reported a prevalence of 0.8–0.9%. Zermiani et al. Collected data from 1997 to 2007 in Verona involving 345 sex workers, with only 1 PWID and an HCV prevalence of 0.9% [46]. In the same period (1999–2008), Prestileo et al. conducted a prospective study in Palermo involving 275 migrant sex workers, with 86.9% of acceptance [47]. Of 239, 2 tested positive, both from Ukraine [47]. Recently, Colpani et al. reported a 20 year-long experience of an outpatient clinic for migrants in Piacenza, with a focus on sex workers’ health. Migrants of different nationalities were included (Africa, Center/South America, Eastern Europe, Asia). Among 936 HCV screening, 6 patients tested positive, with only 1 starting treatment and then being lost to follow-up [48].

Studies on HCV among migrants have been summarized in Table 3.

### 5.3. Clinical Features and Outcomes

A few studies reported genotypes encountered among the migrant population. Scotto et al. analyzed genotypes in 119 patients [36]. The most common was genotype 1 in 58/119 (48.7%), especially found among East-European patients, followed by genotype 4 in 25 (21.0%), mostly African patients. Genotype 2 was found in 13 (10.9%), half of them were Asian patients, while genotype 3 was detected in 23 (19.4%), of whom around 1/3 were Asian patients [36]. No genotype 2 was reported by Prestileo et al., who evaluated only African migrants (44.4% genotype 3, 27.8% 4, 27.8% genotype 1). Similar findings were reported in the penitentiary-based study, with 50% of genotype 3, 14.7% genotype 4, 20.5% genotype 1a, and 8.8% genotype 1b [47].

Regarding clinical presentation, few data are available in the literature. In the study conducted by Scotto et al., 25/119 viremic subjects were staged by biopsy: 13 mild steatoses were reported, 8 moderate to severe, and 4 cases of liver cirrhosis. Participants accepting the biopsy were mainly from East Europe. The same study highlighted similar SVR rates to the general population [36]. The study was conducted in the pre-DAA era, with treatments ranging from 6 to 12 months based on peg-IFN and ribavirin. Consistent data regarding DAAs were reported from the PITER cohort, which also reported no differences in liver injury between native and migrants [43]. On this regard, Prestileo et al. reported no liver cirrhosis among 18 patients evaluated by ultrasound or fibroscan^®^. Eleven of them received treatment, with only one loss to follow-up. The others achieved SVR [49]. The same author reported lower adherence to treatment among foreign inmates (37%) [47].

The modern work-up for HCV staging is much less invasive than a biopsy, requiring only ultrasound, fibroscan^®^, or indirect staging methods, such as FIB-4 or APRI scores. For this reason, the diagnostic tests could be better accepted and the retention in care could be higher than before. Furthermore, DAAs are highly effective, well tolerated, and with very short schedules. Unfortunately, few updated data are available; however, engaging a mobile population exposed to ever-changing life conditions is still difficult. Accurate information on the tolerability and efficacy of treatment and possible long-term complications of an untreated infection could improve the cascade of care of this population [49].

## 6. COVID-19 Influence on HCV Infection Approach

A lack of national literature regarding COVID-19 influence on HCV is available. Surely, as in other prison settings, the Italian system was dramatically affected by the pandemic. The main discussed problem was the overcrowding [50]. During 2020, the Italian prison flow included >96,000 people [50]. For this reason, the majority of the prevention and control strategies were focused on SARS-CoV-2 infection, and not on other diseases, giving access exclusively to the essential personnel and the needing of a wide vaccination was pointed out [51,52,53,54].

Although all isolation measures were activated for the wellbeing of prison population, the result was a limited access by Specialists, decelerating the HCV screening and treatment.

COVID-19 pandemic has rapidly changed the management of HCV [51,52,53,54]. In addition, the disruption of HCV elimination programs during 2020 is anticipated to impact the ability to achieve the World Health Organization (WHO) elimination goals by 2030 [11]. A recently published model demonstrated that a 1-year delay in HCV treatment initiation could result in 746,000 fewer patients starting treatment globally before 2030. The predicted consequence of delaying diagnosis and treatment by one year was an additional 44,800 liver cancers and 72,300 HCV-related deaths by 2030. For the psychiatric population, COVID-19 has further complicated the continuation of psychiatric care with the closure of hospitals and the start of telemedicine, this has caused a decrease in diagnoses and treatments also regarding hepatitis C. Furthermore, COVID-19 has been associated with the unveiling of psychiatric pathologies such as depression and anxiety [55].

The pandemic has certainly affected migrants’ health, perhaps more than what happened for the general population [56]. Many migrants’ outpatient clinics are run by Infectious Diseases specialists, who have been diverted to COVID-19 wards, at least during the critical phases of the pandemic. Thus, undocumented migrants, in particular, could have been left without primary assistance. The events of the last two years have led to missing diagnosing opportunities, treatment delays, and loss to follow-up. The 2030 WHO goals may now be even further apart. This is the time to boost the screening programs, with particular attention to patient information and care retention.

## 7. Discussion and Conclusions

Overall, we found a wide but heterogeneous literature on HCV screening, staging, and treatment in prison settings. Undoubtedly, there is a high spread of viral hepatitis in the Italian prison settings. These data are in line with previous international literature, which highlighted higher HCV prevalence among incarcerated people than in general population [57]. However, increasing studies are demonstrating how it is possible to overcome barriers with quick intervention and short-schedule treatments, particularly when educational programs are provided. This is well known from a couple of years. In 2018, Kronfli et al. demonstrated how counselling meetings at least doubled the HCV screening acceptance in prison population [58]. However, there is still a lack of data about these interventions.

In general, we found a progressive reduction in HCV epidemiology among incarcerated people in the last years, which is actually one-third of what it had been before. Interestingly, data by Voller et al. were the only ones bucking the trend [13]. However, these data came from a single Region, probably reducing the strength when compared with multicenter studies.

More gender-tailored interventions would be useful. In fact, the seroprevalence seems to double when coming to the female population.

Drug use and promiscuous lifestyles are common among psychiatric patients; thus, HCV screening should be considered as part of the clinical routine. Poor compliance to screening programs and treatment uptake, and drug–drug interactions remain open problems in this setting. Interestingly, psychiatric disorders seem to have a bidirectional relationship. In fact, HCV seems to play a role in psychiatric disorder development, and psychiatric disorders have a strong relationship with HCV infection, given the tendence to substances abuse among patients. As an example, a recent review by Gutiérrez-Rojas et al. showed a stratification of HCV prevalence among patients with different disorders. Patients with schizoaffective disorder, schizophrenia, bipolar disorder, major depression, and major depression had a variable HCV seroprevalence ranging from 0.4 to 25%, based on the illness association with lower or higher illicit drugs abuse [59].

In 2021, Girardin et al. reported data on HCV screening among psychiatry inpatients. No data on HCV-RNA positive patients were reported. However, even in this case, the seroprevalence ranged from 10.8% to 25.7% among non-PWIDs and PWIDs, respectively. This confirms the importance of the tendence to illicit drugs use among people suffering from psychiatric disorders, and its influence on HCV epidemiology [60].

Overall, we found an inconsistent assessment of HCV prevalence among migrants.

Furthermore, data are very difficult to compare with international literature. In fact, available studies recorded data from different populations, with different reported prevalence of HCV. Therefore, the reported results need caution in their evaluation.

However, a great acceptance of screening intervention was shown, especially when providing adequate counselling and a continuum in care from first contact to treatment delivery. As suggested by Italian Guidelines published in 2020, a health assessment upon arrival should include HCV screening for people with risk factors or coming from endemic countries. However, no clear indications are given for undocumented migrants established in Europe for more than six months. Therefore, a careful evaluation of risk factors should be conducted; also, migrants should be informed regarding possible routes of transmission and at-risk behaviors. Regarding follow-up, few studies report this data and not always consistently. Again, providing adequate information through a cultural mediator’s assistance should help guarantee adherence to scheduled visits. Another issue is the mobility of this population. While native citizens are provided with electronic sanitary records, which allow a trace of medical history to be kept, undocumented migrants and asylum seekers may lose their previous history.

Certainly, some new national approaches are providing encouraging data on the population of the entire territory. For example, in 2022, the prevention campaign ‘C devi pensare’ started in Emilia Romagna, involving over 1 million people born between 1969 and 1989 (foreigners, incarcerated people, and Public Services for Addictions’ patients included). Thanks to this campaign, among the 240,179 people who underwent screening blood sampling, it was possible to intercept 386 anti-HCV-positive people (prevalence = 0.16%) [61]. A screening among both key populations and birth cohorts (1969–1989) may substantially help to discover unaware individuals [62]. Using the same approach nationally, it would be probably useful as a step towards WHO goals for HCV elimination.

## 8. Limitations of the Study

Several limitations should be addressed regarding our study. Principally, this is a narrative review, and data came from national studies. As a consequence, we have no statistical strength, and the data cannot reflect underserved populations worldwide. A major challenge is surely related to migrants. In fact, available studies include a wide variety of nationalities. This makes it difficult to define the epidemiology in every subpopulation. Regarding genotypes, there is a lack of data in all subpopulations, and we were not able to perform a complete analysis. Furthermore, many studies did not provide data on antibody positivity or prevalence of active infection.

Data included in the paper were restricted to Italy; for this reason, our review may not reflect the international situation of underserved populations. Furthermore, studies included in our review were extremely heterogeneous in their content. This did not allow us to perform a complete analysis on the cascade of care, which was sometimes a missing datum.

Moreover, we focused the attention on prison, psychiatric disorders, and migrants. These are not the only target populations. In fact, homeless and methadone clinics’ patients also need particular attention. In fact, the literature showed how homelessness was highly related with HCV infection when compared to having a stable house [63,64]. Regarding methadone clinics, HCV screening offer is internationally reported to be <70%, with evaluation for HCV treatment in <10% of cases [65,66,67,68,69].

More studies are needed, particularly to assess the actual scenario of HCV among these subpopulations.

## 9. Strength of the Study

To our knowledge, this is the first study aiming to provide on viral hepatitis C among underserved populations in Italy, and focusing on all aspect on the disease, from analyzing screening programs, to report the cascade of care.

## 10. Conclusions

Incarcerated people with HCV infection are young and with low level of fibrosis. As consequence, it would be an extraordinary achievement to stop not only the viral spread, but also the diseases’ progression.

Diagnosing and eradicating HCV among the psychiatric population is possible with meticulous planning and collaboration between Psychiatry Units and Infectious Diseases centers is crucial.

Regarding migrants, there is a lack of measures to guarantee a continuum of care, especially to address chronic diseases such as hepatitis C.

In conclusion, underserved populations still represent the most important targets in HCV micro-elimination.

## Figures and Tables

**Table 1:** Summary of studies reporting HCV epidemiology/treatment in prison population.

Authors	Year	Study Type	Sample (n)	HCV Prevalence (%)	HCV-RNA Positive (%)	Treatment Type	Genotypes (%)	SVR (%)
Babudieri et al. [12]	2005	Prospective cohort	973	38%	90%	NA	NA	NA
Voller et al. [13]	2011	Cross-sectional survey	15,751	9.0%	NA	NA	NA	NA
Sagnelli et al. [14]	2012	Multicenter, prospective cohort	3468	22.8%	NA	NA	NA	NA
Brandolini et al. [19]	2013	Cross-sectional, single-center	695	22.4%	86.5%	IFN	NA	43%
Foschi et al. [15]	2016	NA	468	9.8%	60%	DAAs	1a/1b (48%) 2 (4%) 3 (26%) 4 (10%)	85%
Babudieri. [16]	2016	Multicenter cohort	5353	11.3%	NA	NA	NA	NA
Pontali et al. [20]	2018	Multicenter cohort	142	NP	100%	DAAs	1a (35.9%) 1b (14.8%) 2 (6.1%) 3a (35.9%) 4 (7.3%)	90.8%
Masarone et al. [17]	2020	Monocentric cohort	670	14.05%	79.3%	DAAs	NP	100%
Fiore et al. [18]	2021	Multicenter cohort	2376	10.4%	41%	DAAs	1a (35.6%) 1b (6.9%) 2 (1%) 3a (44.6%) 4 (11.9%)	97.5%

IFN: interferon; DAAs: Direct Acting Antivirals; NA: not available; SVR: sustained virological response.

**Table 2:** Studies reporting HCV data among patients with psychiatric disorders.

Authors	Year	Study Type	Sample (n)	HCV Prevalence (%)	HCV-RNA Positive (%)	Treatment Type	Genotypes (%)	SVR (%)
Raja et al. [25]	2006	Observational	811	2.8%	NA	NA	NA	NA
De Gennaro et al. [26]	2019	Retrospective, single-center	18	NA		DAAs	1a (52.6%) 1b (10.5%) 2 (5.3%) 3 (15.8%) 4 (15.8%)	88%
Dell’Osso et al. [28]	2006	Multicenter controlled pilot study		NA		IFN	1 (68.5%) 2/3 (38.5%)	NA
Boglione et al. [29]	2019	Retrospective	72	NA		DAAs	NA	94.1%
Fiore et al. [27]	2022	Retrospective	297	39.4%	95.2%	DAAs	3a	99.5%

IFN: interferon; DAAs: Direct Acting Antivirals; NA: not available; SVR: sustained virological response.

**Table 3:** Studies reporting HCV data among migrants.

Authors	Year	Type of Study	Sample	Population	HCV Prevalence (%)	HCV-RNA Positive (%)	Treatment	Genotypes (%)	SVR
Zermiani et al. [46]	2012	Retrospective	345	Migrant sex workers	0.9	NA	NA	NA	
Prestileo et al. [41]	2013	Prospective	239	Migrant sex workers	0.8	NA	NA	NA	
Laganà et al. [42]	2015	Prospective, observational, single-center study	320	Pregnant migrant women	0.9	NA	NA	NA	NA
Coppola et al. [34]	2015	Cross-sectional	882	Undocumented, asylum seekers	4.0	83	NA	NA	NA
Scotto et al. [36]	2016	Retrospective, multicenter	3421	Migrants (not specified)	6.2	63	IFN + ribavirine	1 (48.7%) 2 (10.9%) 3 (19.4%) 4 (21%)	48.8%
Prestileo et al. [41]	2017	Model of management	133	Migrant inmates	40.6	63	37%	1a (20.5%) 1b (8.8%) 2 (5.9%) 3 (50%) 4 (14.7%)	NA
Buonfrate et al. [35]	2018	Cross-sectional	118	Asylum seekers	0.85	NA	NA	NA	NA
Cuomo et al. [40]	2018	Retrospective	304	Undocumented, asylum seekers, refugees	3.3	NA	NA	NA	NA
Donisi et al. [38]	2020	Retrospective	315	Asylum seekers	1.9	NA	NA	NA	NA
Marrone et al. [44]	2020	Retrospective	836	Unaccompanied minors	1.1	67	NA	NP	NA
Prestileo et al. [49]	2021	Cross-sectional	2639	Newly arrived undocumented migrants	0.9	75	DAAs	1a (22.3%) 1b (5.6%) 3 (44.4%) 4 (27.7%)	5.6%
Quaranta et al. [43]	2021	Cross-sectional	301	Migrants (not specified)	NA	NA	DAAs	1a (6.6%) 1b (53.5%) 2 (5.2%) 4 (20.5%)	98%
Colucci et al. [33]	2021	Retrospective	362	Newly arrived undocumented migrants	1.7	33	NA	NA	NA
Fiore et al. [37]	2021	Retrospective	62	Refugees	1.6	100	-	NP	0
Fiore et al. [39]	2021	Retrospective	49	Undocumented	2.2	NA	NA	NP	NA
Lapadula et al. [45]	2022	Cross-sectional	66	Migrant transgender sex workers	0	0	NA	NA	

IFN: interferon; DAAs: Direct Acting Antivirals; NA: not available; SVR: sustained virological response.

## Data Availability

All data are available in the manuscript.
